# Heat Stress Affects H3K9me3 Level at Human Alpha Satellite DNA Repeats

**DOI:** 10.3390/genes11060663

**Published:** 2020-06-18

**Authors:** Isidoro Feliciello, Antonio Sermek, Željka Pezer, Maja Matulić, Đurđica Ugarković

**Affiliations:** 1 Department of Molecular Biology, Ruder Bošković Institute, Bijenička 54, HR-10000 Zagreb, Croatia; Antonio.Sermek@irb.hr (A.S.); Zeljka.Pezer.Sakac@irb.hr (Z.P.); 2 Dipartimento di Medicina Clinica e Chirurgia, Universita′ degli Studi di Napoli Federico II, via Pansini 5, I-80131 Napoli, Italy; 3 Department of Biology, Faculty of Science, University of Zagreb, HR-10000 Zagreb, Croatia; maja.matulic@biol.pmf.hr

**Keywords:** alpha satellite DNA, heterochromatin, euchromatin, transcription, heat stress, histone modification, epigenetics, gene silencing

## Abstract

Satellite DNAs are tandemly repeated sequences preferentially assembled into large arrays within constitutive heterochromatin and their transcription is often activated by stress conditions, particularly by heat stress. Bioinformatic analyses of sequenced genomes however reveal single repeats or short arrays of satellite DNAs dispersed in the vicinity of genes within euchromatin. Here, we analyze transcription of a major human alpha satellite DNA upon heat stress and follow the dynamics of “silent” H3K9me3 and “active” H3K4me2/3 histone marks at dispersed euchromatic and tandemly arranged heterochromatic alpha repeats. The results show H3K9me3 enrichment at alpha repeats upon heat stress, which correlates with the dynamics of alpha satellite DNA transcription activation, while no change in H3K4me2/3 level is detected. Spreading of H3K9me3 up to 1–2 kb from the insertion sites of the euchromatic alpha repeats is detected, revealing the alpha repeats as modulators of local chromatin structure. In addition, expression of genes containing alpha repeats within introns as well as of genes closest to the intergenic alpha repeats is downregulated upon heat stress. Further studies are necessary to reveal the possible contribution of H3K9me3 enriched alpha repeats, in particular those located within introns, to the silencing of their associated genes.

## 1. Introduction

Satellite DNAs are tandemly repeated sequences assembled within constitutive heterochromatin at the (peri)centromeric and subtelomeric regions [1]. However, some satellite DNAs are not only clustered within heterochromatin in the form of long arrays but are dispersed as short arrays within euchromatin, in the intronic and intergenic regions [2,3,4,5,6]. Although satellite DNAs are preferentially embedded in constitutive heterochromatin which is considered transcriptionally inert, their transcription was reported in vertebrates, invertebrates, and plants [7]. Constitutive heterochromatin is sensitive to temperature fluctuations and is dynamically regulated in response to environmental stimuli [8,9]. Temperature-mediated heterochromatin disruption seems to be an epigenetic event which includes stress-response transcription factors involved in heterochromatin assembly. In human cells, heat stress activates heat shock transcription factor 1 (HSF1) which recruits major cellular acetyltransferases to pericentric heterochromatin leading to targeted hyperacetylation [10], facilitating particularly the transcription of satellite III DNA [11,12]. In *Drosophila*, under standard conditions, transcription factor dATF-2, which regulates expression of stress response genes, recruits heterochromatin protein 1 (HP1) to pericentromeric heterochromatin, while under heat stress, activated MAP kinases such as p38 phosphorylate dATF-2 which is released from heterochromatin, leading to abolishment of HP1 and disruption of heterochromatin [13]. In vivo studies on the insect *Tribolium castaneum* revealed heat-stress induced destabilization of heterochromatin coupled with overexpression of a major heterochromatic satellite DNA TCAST1, followed by processing of satellite transcripts into siRNAs [14]. Satellite DNA-derived small interfering RNAs are, through RNA interference mechanism (RNAi), involved in the epigenetic process of heterochromatin formation in different organisms [15,16,17]. In *T. castaneum*, heat-stress induced transcription of TCAST1 satellite DNA correlates with an increased level of repressive heterochromatin marks H3K9me2/3 not only on satellite repeats in constitutive heterochromatin but also on dispersed TCAST1 satellite elements and their proximal regions within euchromatin, affecting expression of nearby genes [18]. 

Alpha satellite DNA comprises up to 10% of the human genome and is located in the centromeric and pericentromeric regions of all chromosomes [19]. The fundamental unit of human alpha satellite DNA is based on diverged 171 bp monomers which are often organized in complex higher order repeats [20]. In addition to their (peri)centromeric location, a bioinformatic search of the human genome revealed the presence of 133 blocks of alpha satellite located > 5 Mb from the centromere [21]. Alpha satellite DNA is actively transcribed and transcripts contribute to essential chromosomal functions such as centromere and kinetochore assembly and heterochromatin formation [19], while interaction between SUV39H1 histone methyltransferase and pericentromeric alpha satellite DNA transcripts is necessary for proper heterochromatin silencing in humans [22].

In this paper, using different human cell lines as a model system, we analyze transcription of alpha satellite DNA upon heat stress. In addition, we followed epigenetic changes of histones, in particular the “silent” H3K9me3 and “active” H3K4me2/3 marks at alpha repeats dispersed within euchromatin, in introns and at intergenic regions as well as tandemly arranged alpha satellite which are characteristic for heterochromatin. The results reveal an increase of H3K9me3 level at alpha repeats and spreading of H3K9me3 to the flanking regions up to 1-2 kb from the insertion sites of the euchromatic repeats. The dynamics of H3K9me3 enrichment at alpha repeats correlates with the dynamics of alpha satellite DNA transcription activation upon heat stress, being the most prominent immediately after heat stress. No change in the level of H3K4me2/3 is detected either at heterochromatic or euchromatic alpha satellite repeats. We also followed the dynamics of expression of genes containing alpha repeats within introns as well as of the genes closest to the intergenic alpha repeats, upon heat stress. Expression of the genes is downregulated, being negatively correlated with H3K9me3 levels at the associated alpha repeats. Further studies are necessary in order to reveal the possible contributions of H3K9me3 enriched alpha repeats, in particular those located within introns, to the silencing of their associated genes.

## 2. Materials and Methods 

### 2.1. Bioinformatic Analysis

Alpha satellite repeats annotated in the human genome assembly GRCh17.hg19 were extracted from the rmsk table in the UCSC Table Browser. Organization of repetitive sequences identified by RepeatMasker at regions near dispersed alpha repeats was analyzed by UCSC Genome Browser.

### 2.2. Human Cell Lines

The following human cell lines were used in the experiments: MJ90 hTERT (immortalized skin fibroblasts), 697 (human Pre-B leukemia), MCF-7 (human breast adenocarcinoma), HT29 (colon cancer adenocarcinoma), Cal 27 (oral cancer adenosquamous), HeLa (human cervix carcinoma), Hep2 (human cervix carcinoma HeLa derivative), SW620 (colon adenocarcinoma), SW48 (colorectal adenocarcinoma), DLD1 (colorectal adenocarcinoma), HCT116 (colon carcinoma), ZR75 (breast carcinoma), HepG2 (liver hepatocellular carcinoma), A431 (squamous carcinoma), 293T (human embryonic kidney), and OV-90 (ovarian adenocarcinoma). Cells were cultured in appropriate medium (DMEM or RPMI1640) supplemented with 10% FBS and 5% CO_2_ at 37 °C. For heat shock experiments cells were incubated for 1 or 2 h at 42 °C in complete medium, followed by recovery at 37 °C. Chaetocin from Abcam was used as a specific inhibitor of the human histone methyltransferase Suv39H1.

### 2.3. DNA Extraction and PCR Analysis in Cell Cultures

Genomic DNA was prepared using the “GenElute Mammalian Genomic DNA Miniprep Kit” Sigma-Life Science. PCR reactions were performed using 2× DreamTaq Green PCR Master Mix (Fermentas) in a final reaction volume of 20 μL containing 0.2 μM of each primer and 50 ng of genomic DNA. Primers used in the analysis of insertion polymorphism of dispersed alpha satellite repeats were designed in a unique sequence around the insert using Primer3Plus software. Primers are listed in Table S1. The reaction conditions used for the amplification were: 94 °C for 1 min, 10 cycles at 94 °C for 30 s, 60 °C for 30 s (with annealing temperature declining in each cycle for 0.25 °C) and 72 °C for 1 min followed by 20 cycle at 94 °C for 30 s, 55 °C for 30 s, and 72 °C for 90 s. PCR amplification products were separated by agarose gel electrophoresis, isolated from gel using QIAquick gel extraction kit and sequenced using an ABI Prism 310 (Applied Biosystems).

### 2.4. RNA Isolation and Reverse Transcription

For RNA isolation from cell cultures lysis buffer was added directly after the PBS washing step avoiding trypsin treatment. RNA was additionally digested with Turbo DNase (Ambion) and quantified with the Quant-IT RNA assay kit using a Qubit fluorometer (Invitrogen). Integrity of RNA was checked by gel electrophoresis and the presence of DNA contamination was checked by PCR. Approximately 1 μg of RNA was reverse transcribed using the PrimeScript RT reagent Kit with gDNA Eraser (perfect Real Time, Takara) in 10 μL reaction using random and oligo dT primers mix. For all samples negative controls without reverse transcriptase were used. 

### 2.5. Quantitative Real-Time PCR (qPCR) Analysis

qPCR analysis was performed according to the previously published protocol [18]. Primers for the expression analysis of human alpha satellite DNA and of genes associated with alpha repeats are listed in Table S2. *Glucuronidase beta (GUSB*) [23] was used as an endogenous control for normalization in human samples. *GUSB* gene (Gene ID: 2990) was stably expressed without any variation among samples after heat stress. The following thermal cycling conditions were used: 50 °C 2 min, 95 °C 7 min, 95 °C 15 s, 60 °C 1 min for 40 cycles followed by dissociation stage: 95 °C for 15 s, 60 °C for 1 min, 95 °C for 15 s, and 60 °C for 15 s. Amplification specificity was confirmed by dissociation curve analysis and specificity of amplified products was tested on agarose gel. Control without template (NTC) was included in each run. Post-run data were analyzed using LinRegPCR software v.11.1. [24,25] which enables calculation of the starting concentration of amplicon (“No value”). No value is expressed in arbitrary fluorescence units and is calculated by taking into account PCR efficiency and baseline fluorescence. “No value” determined for each technical replicate was averaged and the averaged “No values” were divided by the “No values” of the endogenous control. Statistical analysis of qPCR data was done using GraphPad v.6.01 and normalized No values were compared using the unpaired t-test which compares the mean of two unmatched groups.

### 2.6. Chromatin Immunoprecipitation

MJ90 hTERT, Cal 27, HepG2, and OV-90 cells were grown to subconfluence, washed in PBS, scraped in nuclear isolation buffer (10 mM MOPS; 5 mM KCl; 10 mM EDTA; 0.6% Triton X-100) with protease inhibitor cocktail CompleteMini (Roche) and chromatin immunoprecipitation was performed according to the published protocol [18]. The antibodies used were H3K9me3 (Abcam, ab8898), H3K4 di + tri methyl (Abcam, ab6000), and IgG (Santa Cruz Biotechnology, sc2027). Binding of precipitated target was monitored by qPCR using the SYBR Green PCR Master mix. Primers for polymorphism analysis listed in Table S1 were used to check the H3K9me3 level at alpha repeats under reaction conditions described above. Primers used for H3K9me3 level analysis at regions distant to alpha repeats are also listed in Table S1 while primers for alpha satellite, NR3C1-HepG2+ and NR3C1-HepG2- are listed in Table S2. The No value was normalized using No value of input fraction.

## 3. Results

### 3.1. Dispersion Profile and Polymorphism of Dispersed Alpha Satellite Repeats

Alpha satellite repeats (ARs) annotated in the human genome were downloaded using the UCSC table browser. The human reference genome GRCh38.hg38 was also searched with the alignment program BLASTN version 2.3.1+ using as the query a dimer of human alphoid 171 bp repeat consensus sequence derived from 293 cloned monomers of wide-ranging chromosomal origins [26]. The analyses revealed preferential organization of alpha satellite DNA within clusters composed of tandemly arranged repeats and the largest clusters are located within centromeric and pericentromeric regions of all chromosomes. A search for alpha repeats in chromosomal regions outside of (peri)centromeres revealed their presence not only in long clusters but also as short arrays of 1–4 monomers, located at intergenic regions or within introns. Only hits with at least 50% of 171 bp monomer sequence length were considered for further analysis. We detected in total 31 short dispersed alpha satellite DNA elements, mostly partial or full size monomers (Table S3). Ten of the elements are within introns while the remaining 21 are in intergenic regions and the genes closest to the intergenic alpha repeats from 5′ and 3′ site are listed in Table S3. Some of the alpha repeats are located between the same genes (No 11 and 12; 22 and 23; 26 and 27), and the total number of genes that are associated with intronic or intergenic alpha repeats is 46 (Table S3). 

We first decided to analyze the insertion polymorphism of short dispersed alpha satellite among 16 different human cell lines. Almost all dispersed alpha repeats have in the vicinity other repetitive elements such as *Alu*, L1, or LTR and for some alpha repeats flanking repetitive sequence prevented construction of single locus-specific primers. Analysis of insertion polymorphism was finally performed for all 10 alpha satellite elements located within introns as well as for 10 elements in intergenic regions (Table S3). Out of 20 tested dispersed alpha satellite elements only two revealed insertion polymorphisms: element No 29 located within an intron of *zinc finger protein 675* gene (*ZNF675*) and element No 10 located within intron of *nuclear receptor subfamily 3 group C member* 1 gene (*NR3C1*) (Table S3). Element No 29 corresponds to 0.6 of alpha satellite monomer length and is homozygous in all tested cell lines except Cal 27 and OV-90 where it is absent on both alleles (Figure S1A,B). Alpha element No 10 corresponds to a 1.1 satellite monomer and is heterozygous in the HepG2 cell line being absent on one allele as revealed by sequencing (Figure S1C), while in all other tested cell lines it is homozygous. 

### 3.2. Alpha Satellite DNA Transcription after HS

**Figure 1:**
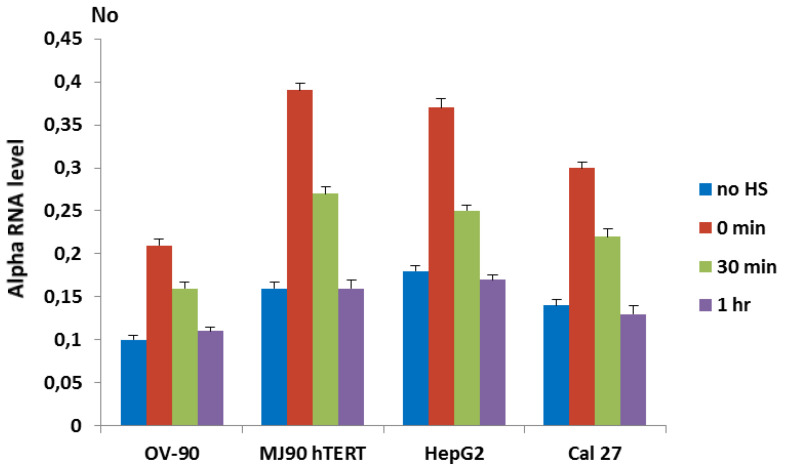
The dynamics of transcription of alpha satellite DNA in cell lines OV-90, MJ90 hTERT, HepG2 and Cal 27 under standard conditions (no HS), immediately after 2 h of heat stress at 42 °C (0 min), at 30 min and 1 h of recovery at 37 °C, respectively. Two experiments were performed on each cell line. No represents normalized average No value. Columns show average of two different RT-qPCR experiments performed in triplicate and error bars represent standard deviations.

To investigate if heat stress (HS) affects transcription of alpha satellite DNA we followed its transcription dynamics in human cell lines by RT-qPCR under standard and heat stress conditions. Primers used for transcription analysis were constructed based on consensus sequence derived from cloned alpha satellite monomers of wide-ranging chromosomal origins [26] and were able to amplify only tandemly arranged repeats (Figure S2). Since in human pericentromeric heterochromatin alpha satellite DNA is organized in tandemly arranged monomers [19], it can be expected that the primers preferentially recognize transcripts deriving from pericentromeric regions. For heat shock experiments, cells were incubated for 1 or 2 h at 42 °C in complete medium, followed by a recovery period at 37 °C. The transcription of alpha satellite DNA was checked immediately after heat stress as well as after a recovery period of 30 min and 1 h, and was compared with a control that was not subjected to heat stress. The transcription of alpha satellite DNA was monitored in immortalized fibroblasts (MJ90 hTERT), oral cancer adenosquamous cell line (Cal 27), ovarian adenocarcinoma cell line (OV-90), and liver hepatocellular carcinoma cells (HepG2). The analysis of transcription revealed differences in alpha RNA level among cell lines at standard conditions. However, the dynamics of transcription after HS is similar in all cell lines (Figure 1). The level of alpha satellite RNA is increased from 2.0× in OV-90 cells to 2.4× in MJ90 hTERT (*p* < 0.05) relative to the controls (no HS) immediately after 2 h of HS. The level of alpha RNA is decreased during the recovery period of 30 min at 37 °C, while after 1 h of recovery it drops to the level of control (Figure 1). 

### 3.3. Dynamics of H3K9me3 at Alpha Satellite Repeats after HS

The enrichment of repressive histone marks at dispersed repeats of a major satellite DNA and their neighboring regions after HS was previously demonstrated in the beetle *T. castaneum* [18]. Here we analyze distribution of the silent histone mark H3K9me3 at dispersed alpha satellite repeats and flanking regions both at standard conditions and after heat stress. We performed chromatin immunoprecipitation (ChIP) coupled with quantitative real-time PCR, using specific primers (Table S1). H3K9me3 levels were analyzed at 18 dispersed alpha satellite elements and their flanking regions, 10 of them located within introns and 8 at intergenic regions. Using the same method we also analyzed the level of H3K9me3 at regions located within 0.5–6 kb distance to each of the AR. ChIP assay was performed on chromatin isolated from MJ90 hTERT, Cal 27, OV-90, or HepG2 cells subjected to a heat shock of 2 h at 42 °C. The level of H3K9me3 was measured immediately after HS and at 30 min post-recovery at 37 °C, and was compared to the level of control (no HS) using the unpaired *t*-test. In addition, we followed the dynamics of binding of IgG to dispersed alpha satellite repeats and tandemly repeated satellite arrays and the amount of bound IgG was very low resulting in a signal below the qPCR threshold. 

**Figure 2:**
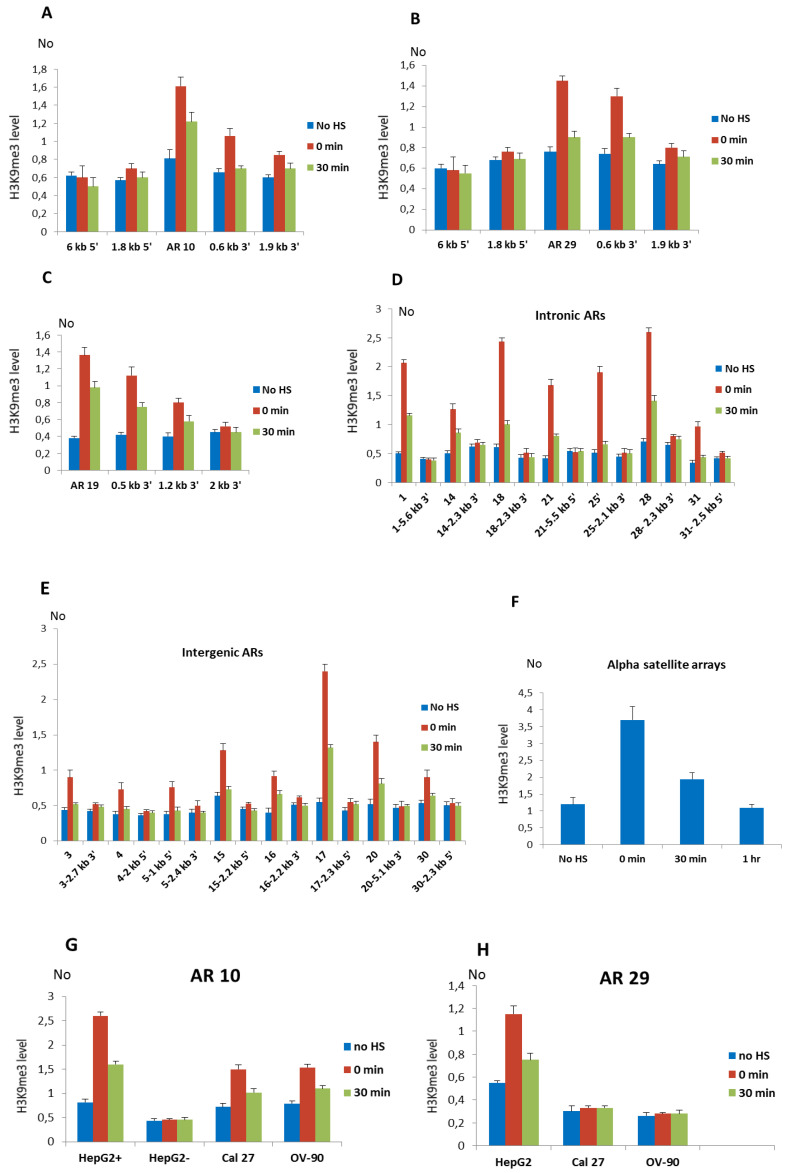
The level of H3K9me3 at alpha satellite repeats (ARs) and their proximal regions. (**A**) The profiles of H3K9me3 distribution in MJ90 hTERT cells at alpha repeat 10, (**B**) alpha repeat 29, (**C**) alpha repeat 19, and at their neighboring regions positioned 0.5–6 kb to each of the AR. (**D**) H3K9me3 level at intronic alpha satellite repeats No 1, 14, 18, 25, 21, 28, 31, and (**E**) intergenic alpha satellite repeats No 3, 4, 15, 16, 17, 20, and 30 as well as at the region 1 kb 5′ to intergenic AR No 5. In addition, H3K9me3 level at the regions positioned 2–6 kb from each of the alpha repeat, either at 5′ or 3′ site is shown. (**F**) H3K9me3 level at tandemly arranged alpha satellite repeats characteristic for heterochromatin and at (**G**) AR 10 in intron of *NR3C*1 gene in Cal 27 and OV-90 cells as well as in HepG2 cells on allele with (HepG2+) and without AR (HepG2-), (**H**) AR 29 in intron of *ZNF675* gene in HepG2 cells as well as in the corresponding intron region without alpha satellite repeat in Cal 27 and OV-90 cells. H3K9me3 levels were measured by ChIP coupled by quantitative real-time PCR at standard conditions (no HS), immediately after 2 h of HS (0 min), at 30 min (30 min) and 1 h of recovery. No value was normalized using No value of input fraction and represents the H3K9me3 level. Columns show average of three independent experiments and error bars indicate the standard deviations.

The profile of H3K9me3 distribution after HS at alpha repeats and their neighboring regions was first examined at intronic ARs No 10 (1.1 monomer), 19 (2.4 monomers), and 29 (0.6 monomer) in MJ90hTERT cells using specific primers. The primers used amplified regions encompassing ARs as well as several regions, approximately 200 bp long, positioned 0.5–6 kb from the corresponding AR (indicated as red horizontal bars in Figure S3). The H3K9me3 level was increased 2.1× at the AR 10 immediately after HS and remains increased 1.5× after 30 min of recovery (Figure 2A). At a region positioned 656 bp 3′ to AR 10, 1.7× (*p* = 0.011) increase of H3K9me3 was detected immediately after HS while no significant change in H3K9me3 level was detected at regions located 1.8 kb 5′, 1.9 kb 3′, and 6 kb 5′ to AR 10 (Figure 2A). Since a region 6 kb distant to AR 10 encompasses part of a L1 repeat while a 1.8 kb distant region at 5′ end flanks an *Alu* repeat (Figure S3), an unchanged H3K9me3 level at these regions suggests no H3K93 enrichment on L1 and *Alu* repeats. The profile of H3K9me3 distribution at the AR 29 is characterized by an increase of 1.9× and 1.3× relative to the control immediately after HS and at 30 min of recovery, respectively (Figure 2B). At a region 625 bp 3′ to AR 29, H3K9me3 level was increased 1.6× (*p* = 0.009) immediately after HS, while at more distant regions positioned 1.8 kb 5′ and 1.9 kb 3′ to AR a statistically insignificant increase of H3K9me3 level was observed as well as no change at 6 kb distance (Figure 2B). At AR 19, the increase of H3K9me3 immediately after HS was 3.6×, while at distances of 0.5 kb and 1.2 kb, the H3K9me3 level increased 2.7× and 2× respectively, while at 2.4 kb distance the level was close to the control (Figure 2C). The results reveal that the H3K9me3 profiles are characterized by a maximal increase at ARs and spreading of H3K9me3 to nearby regions up to approx. 1–2 kb (Figure 2A–C). 

The level of H3K9me3 after HS was also examined at the seven remaining alpha satellite repeats located within introns of genes: ARs No 1, 14, 18, 21, 25, 28, and 31 (Figure 2D). The H3K9me3 mark was also examined at the intronic regions positioned 2–6 kb from each of the AR (Figure 2D). It was expected that other ARs also exhibit spreading of H3K9me3 up to 1–2 kb to the neighboring regions making therefore 2–6 kb ARs′ distant regions suitable as negative controls. In the same way we examined H3K9me3 level at intergenic ARs No 3, 4, 15, 16, 17, 20, and 30 as well as at the regions positioned 2–6 kb from each of the intergenic AR (Figure 2E). For intergenic AR 5 which is composed of a 3.4 mer (Table S3) and due to its size cannot be efficiently amplified by qPCR, we examined H3K9me3 level at the regions positioned 1 kb 5′ and 2.4 kb 3′ to AR, respectively (Figure 2E). The H3K9me3 level at all ARs, either intergenic or intronic, is characterized by an increase ranging from 1.7× for the intergenic AR No 30 to 4.3× for the intronic AR No 18 relative to the control, immediately after HS (*p* < 0.05; Figure 2D,E; Table 1), while after 30 min of recovery the level of H3K9me3 decreases at all ARs. The H3K9me3 level at the region positioned 1 kb 5′ to AR No 5 is increased 1.8× immediately after HS (Figure 2E). No significant correlation between size of either intergenic or intronic ARs and their H3K9me3 enrichment upon heat stress (Table 1) is found. At regions 2-6 kb distant to ARs no significant change in H3K9me3 level was observed upon heat stress (Figure 2D,E), confirming similar spreading of H3K9me3 from all ARs after HS which is up to maximally 2 kb to the nearby region. We also followed the level of H3K9me3 at tandemly arranged alpha satellite repeats characteristic for heterochromatin (Figure 2F). In MJ90 hTERT cells H3K9me3 levels increased 3.2× (*p* = 0.009) immediately after HS while at 30 min of recovery the level decreases and drops to the level of the control at 1h of recovery (Figure 2F). In addition to MJ90hTERT cells, we also examined the H3K9me3 level at intronic ARs in a HepG2 cell line and the results revealed a significant increase of H3K9me3 of 2–3.4× (*p* < 0.05) after HS on all ARs while no significant change was observed at the regions 2–6 kb distant to ARs which served as negative controls (Figure S4). 

For two intronic ARs No 10 and 29 which exhibit an insertion polymorphism (Figure S1) we analyzed H3K9me3 levels at the corresponding intronic regions in cell lines where these ARs are absent. In HepG2 cells alpha repeat No 10 is heterozygous, and to follow H3K9me3 levels separately at each allele, we used allele specific primers (see Figure S1C). At the allele with AR No 10, the H3K9me3 level at the AR and nearby region was increased 3.2× and 1.9× after HS and at 30 min of recovery, respectively, while at the allele without alpha repeat No 10 we did not detect a change in H3K9me3 level at the corresponding intronic region of *NR3C1* gene (Figure 2G). The increase of H3K9me3 levels at the alpha repeat No 10 was also detected immediately after HS in Cal 27 (2.1×) and OV-90 (1.7×) cells (Figure 2G). The H3K9me3 level at the alpha repeat No 29 was increased 2.1× immediately after HS in HepG2 cells, while in Cal 27 and OV-90 cells where the *ZNF675* gene does not contain an alpha repeat (Figure S1B), no significant change in H3K9me3 level after HS at a corresponding intron region is detected (Figure 2H). Since the *Alu* repeat in which AR 29 is imbedded remains in Cal 27 and OV-90 cells (Figure S1B), the absence of H3K9me3 change at the corresponding region, without AR 29 shows that the alpha repeat and not *Alu* is responsible for H3K9me3 enrichment upon HS. Finally, we followed the level of “active” histone marks H3K4me2/3 at tandemly arranged and dispersed ARs upon HS. No significant change in H3K4me2/3 level is detected immediately after HS and at 30 min of recovery (*p* > 0.05; Figure S5).

The results reveal H3K9me3 enrichment at dispersed ARs and spreading to the flanking regions up to 1–2 kb after exposure of different human cells to heat shock, while in the absence of ARs no change in the H3K9me3 level is observed at the corresponding regions. In addition, the dynamics of pericentromeric alpha satellite DNA transcription after HS (Figure 1) correlates with an increase of H3K9me3 at dispersed and tandemly arranged satellite repeats (Figure 2), suggesting a role for alpha satellite DNA transcripts in the enrichment of repressive histone marks at satellite repeats, and is in accordance with previous work on TCAST1 satellite DNA [14,18].

### 3.4. Expression of Alpha Repeat-Associated Genes after HS

Among genes associated with 20 dispersed alpha satellite repeats previously tested for insertion polymorphism (Table S3), we selected those showing a reliable level of expression by qPCR in at least one cell line. Among genes that have alpha satellite elements located in introns, three genes (*PLA2G12B, DLG2, MAP7D2*) were expressed at a very low level while the seven remaining genes were suitable for expression study: *NR3C1*, *ZNF675*, *VAV1, SLC30A6, PRIM, STAM,* and *MYO1E*. Finally, we also tested expression of 14 genes closest to the intergenic alpha repeats, which are up to 50 kb away from the intergenic ARs (Tables S2 and S3), and eight of them were suitable for expression study (*SLC40A1, ASNSD1, ST6GAL1, HTRA3, ACOX3, INTS1, PHF20L1,* and *DIP2C*). 

**Figure 3:**
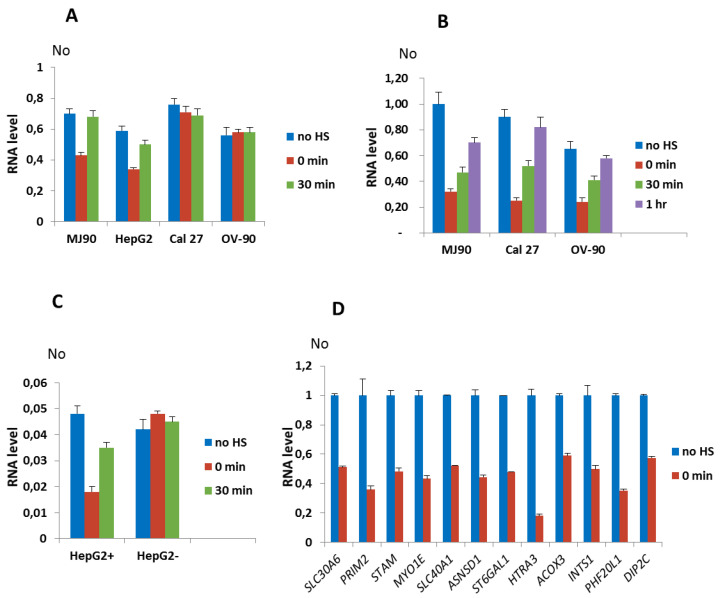
Dynamics of expression of genes associated with dispersed alpha satellite repeats after HS. Expression of: (**A**) *ZNF675* associated with polymorphic alpha satellite repeat 29 which is present in cell lines MJ90 hTERT and HepG2, and absent in Cal 27 and OV-90, under standard conditions (no HS), immediately after 2 h of HS (0 min) and 30 min of recovery, (**B**) *NR3C1* in cell lines MJ90 hTERT, Cal 27 and OV-90 where alpha repeat 10 is present on both alleles, and (**C**) *NR3C1* allele specific expression in HepG2 cells from the allele with (HepG2+) and without (HepG2-) alpha repeat 10, under standard conditions (no HS), after 2 h of heat stress (0 min), and at 30 min and 1 h of recovery, (**D**) *SLC30A6, PRIM2, STAM,* and *MYO1E* with alpha repeats in intron and *SLC40A1, ASNSD1, ST6GAL1, HTRA3, ACOX3, INTS1, PHF20L1, DIP2C* associated with intergenic alpha repeats. Expression is analyzed in MJ90 hTERT cell line under standard conditions (no HS) and immediately after 2 h of HS (0 min). In (**A**–**C**) No represents normalized average No value for each gene. In (**D**) relative No values are shown that are obtained by dividing each No value by No value of control (no HS), for each gene. Columns show average of two different RT-qPCR experiments performed in triplicate and error bars represent standard deviations.

We first tested expression of genes associated with ARs that showed insertion polymorphism such as *ZNF675* and *NR3C1* genes. The dynamics of expression of the *ZNF675* gene in MJ90 hTERT fibroblasts and in HepG2 cells is characterized by a downregulation of 1.7× relative to the control immediately after 2 h of HS in both cell lines, while in Cal 27 and OV-90 cell lines where *ZNF675* is not associated with an alpha satellite repeat, the expression was not affected by HS (Figure 3A). The maximal downregulation of *NR3C1* expression relative to the control was observed immediately after 2 h of HS in MJ90 hTERT (3.5×), Cal 27 (3.7×), and OV-90 (2.7×) cells and is followed by an increase in expression after recovery periods of 30 min and 1 h, respectively (Figure 3B). Since in HepG2 cells the alpha repeat within the intron of *NR3C1* gene is heterozygous, we used allele-specific primers (Figure S1C) for the expression analysis. *NR3C1* expression from the allele containing an alpha repeat was downregulated 2.7× immediately after HS, followed by an increase in expression after 30 min of recovery, while *NR3C1* expression from the allele without the alpha repeat was not affected by HS (Figure 3C). 

The results reveal transient downregulation of *NR3C1* and *ZNF675* genes associated with alpha repeats after HS in different cell lines, while in the absence of the alpha repeat no effect on the expression of these genes is observed. The dynamics of H3K9me3 accumulation at ARs 10 and 29 after HS (Figure 2A,B,G,H) correlates with the suppression of AR 10 and AR 29-associated genes *NR3C1* and *ZNF675*, respectively, after HS (Figure 3A–C), indicating a possible influence of H3K9me3 enrichment at the ARs on gene expression downregulation.

The expression analysis of genes *SLC30A6, PRIM2, STAM,* and *MYO1E* with alpha satellite elements located with introns as well as of eight genes associated with intergenic alpha satellite elements was measured immediately after 2 h of HS at 42 °C in the MJ90 hTERT cell line (Figure 3D). The expression of the *VAV1* gene was followed in the 697 cancer human Pre-B leukemia cell line because in MJ90 hTERT fibroblasts the gene is expressed at very low levels (Figure S6). HS was performed for 1 h since 697 cells do not completely survive longer HS. The decrease in gene expression after HS is observed for all tested genes (Figure 3D and Figure S6). The level of downregulation with statistical significance expressed in P values, calculated by the unpaired t-test, is listed in Table 1 for all tested genes as well as the size and position of gene-associated alpha satellite elements, their distance from the transcription start site (TSS) and cell lines used in the expression analyses. The suppression level of alpha repeat-associated genes is within a range from 1.7× for *ZNF675* and *ACOX3* to 5.6× for *HTRA3*. The distance of dispersed alpha satellite elements from the TSS of genes as well as the distance of intergenic repeats from 5′ or 3′ gene ends, respectively, is not significantly correlated with the level of gene suppression (*p* values > 0.05).

### 3.5. Influence of Suv39H1 Inhibition on Expression of Alpha Repeat-Associated Genes after HS

To study the possible role of silent histone modification H3K9me3 on suppression of alpha repeat-associated genes we used chaetocin, a specific inhibitor of the histone methyltransferase SU(VAR)3-9 of *Drosophila melanogaster* and of its human ortholog Suv39H1 [27]. Suv39H1 is a key enzyme in establishing condensed heterochromatin by specifically di- and trimethylating Lys9 of histone H3 and the specificity of chaetocin for the enzyme makes this compound an excellent tool for the study of heterochromatin-mediated gene suppression. 

**Figure 4:**
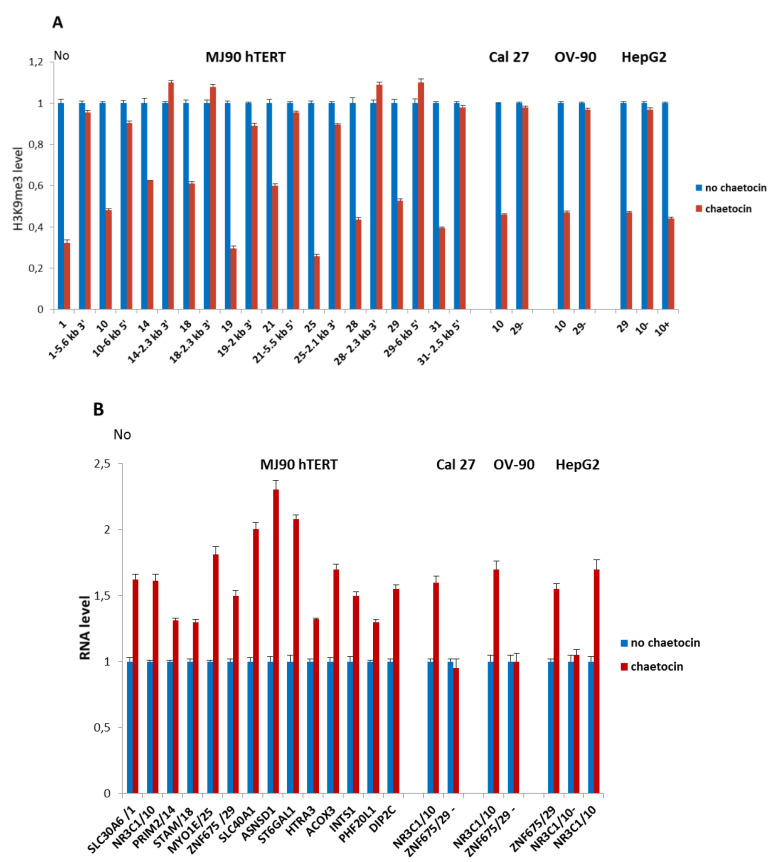
(**A**) The level of H3K9me3 at alpha satellite repeats and their neighboring regions after HS in the presence of chaetocin, inhibitor of histone methyltransferase Suv39H1. H3K9me3 level at intronic alpha satellite repeats No 1, 10, 14, 18, 19, 25, 21, 28, 29, 31 and at the regions positioned 2–6 kb from each of the alpha repeat, either at 5′ or 3′ site is presented in MJ90 hTERT cells. In Cal 27, OV-90, and HepG2 cells, H3K9me3 level was shown for polymorphic ARs 10 and 29. AR 29 is absent in Cal 27 and OV-90 cells (29-), while AR 10 is absent on a single allele in HepG2 cells (10-). (**B**) Expression of genes associated with dispersed alpha satellite repeats after HS in the presence of chaetocin. Genes associated with six intronic ARs: 1, 10, 14, 19, 25, and 29, and with eight intergenic ARs are expressed in MJ90 hTERT cells. Expression of genes *ZNF675* and *NR3C1* which are associated with polymorphic ARs 29 and 10, respectively, is also examined in Cal 27 (AR 29-), OV-90 (AR 29-), and HepG2 (AR 10-) cells. Cells were treated with 150 µM chaetocin during 2 h of heat stress at 42 °C. H3K9me3 level and gene expression were measured immediately after HS and compared with the controls without chaetocin. H3K9me3 was measured by ChIP-qPCR, No values were normalized using No value of input fraction and relative No values are shown which are obtained by dividing each No value by No value of control (no chaetocin). Expression of genes is shown in relative No values which are obtained by dividing each No value by No value of control (no chaetocin), for each gene. Columns show average of two different qRT-PCR experiments performed in triplicate and error bars represent standard deviations.

MJ90 hTERT cells were incubated in complete medium and chaetocin was added to a concentration of 150 µM [28] immediately before HS. In order to check if chaetocin under such conditions affects H3K9me3 level at ARs, we examined H3K9me3 levels at intronic ARs immediately after 2 h HS in the presence and in the absence of chaetocin (Figure 4A). As controls, we examined H3K9me3 levels at regions positioned 2–6 kb from ARs. The results reveal a significant decrease of H3K9me3 levels on all ARs ranging from 1.6× for ARs No 14 and 18 to 3.9× for AR No 25 (*p* < 0.05). No significant change in the H3K9me3 level was detected at regions 2–6 kb distant to ARs (Figure 4A; *p* > 0.05). In the same way we tested the effect of chaetocin on H3K9me3 level at ARs 10 and 29 in Cal 27, OV-90, and HepG2 cells, respectively, where these ARs exhibit insertion polymorphism. In the absence of the corresponding ARs (AR 10-, AR 29-) no change of H3K9me3 level was observed (Figure 4A). 

We were interested to check if the observed decrease of H3K9me3 at ARs reflects on the expression of their associated genes. Expression of six genes with intronic ARs (*SLC30A6, NR3C1, PRIM2 STAM, MYO1E,* and *ZNF675*) and eight genes associated with intergenic ARs (*SLC40A1, ASNSD1, ST6GAL1, HTRA3, ACOX3, INTS1, PHF20L1,* and *DIP2C*) was measured by qRT-PCR after HS in the presence and in the absence of chaetocin (Figure 4B). An increase in the expression of all genes in MJ90 hTERT cells treated with chaetocin relative to the controls without chaetocin treatment was observed (Figure 4B; Table S4). Using the same conditions we monitored expression of genes *ZNF675* and *NR3C1* associated with polymorphic ARs 29 and 10 respectively, in cell lines Cal 27, OV-90, and HepG2. Expression of *ZNF675* gene was not affected by chaetocin in cell lines Cal 27 and OV-90 where AR 29 is absent. *NR3C1* expression from the allele with the AR 10 was increased, while *NR3C1* expression from the allele without the alpha repeat was not affected by chaetocin in HepG2 cells (Figure 4B; Table S4).

The results show that the decrease of H3K9me3 level at ARs is coupled with the enhanced expression of alpha repeat-associated genes. In the absence of ARs, expression of two of these genes, *ZNF675* and *NR3C1*, is not affected by chaetocin, indicating that H3K9me3 enrichment at ARs might contribute to expression downregulation of these two genes after HS. 

## 4. Discussion

Transcription of heterochromatic satellite DNAs is activated by heat stress in different organisms, from plants [29,30] and insects [14,31] to humans [11,12]. In particular, transcription of human pericentromeric satellite III which binds HSF1 is drastically increased during and after HS, exceeding controls by 10–40× [32]. On the other hand, induction of transcription of alpha satellite DNA by HS is much lower relative to satellite III, as observed previously in some cell lines [32]. In agreement with this observation, we detected 2× overexpression of alpha satellite DNA exceeding control, immediately after heat stress in different cell lines. What is the potential function of stress induced transcription of satellite DNA? It was shown that human satellite III transcripts mediate the recruitment of a number of RNA binding proteins involved in pre-mRNA processing and participate in the control of gene expression upon heat stress, at the level of splicing regulation [33]. Transcripts of many satellite DNAs are necessary for heterochromatin establishment [34] and increased satellite transcription after heat stress correlates with H3K9me3 enrichment at tandemly arranged satellite repeats, as shown previously for a major TCAST1 satellite DNA [13] and here for a major human alpha satellite DNA. It was proposed that increased TCAST1 satellite transcription reinforces “heterochromatinization” and helps heterochromatin recovery upon heat stress [14]. Since heterochromatin is important for genome stability and integrity, satellite DNA transcripts might have protective effects in stressed cells/organisms. Centromeric satellite DNA transcripts also play a structural role in centromere or kinetochore integrity in many species including humans and beetles [35,36] and their accumulation under stress conditions seems to be a conserved feature of the cellular stress response [37]. In addition to H3K9me3 enrichment within heterochromatin, the collateral “heterochromatinization” at satellite repeats dispersed within euchromatin occurs, as shown previously for TCAST1 satellite and here for human alpha satellite DNA. Aside from satellite DNA, some transposons in plants [38], *Drosophila* [39,40] and mammals [41,42] are enriched for repressive epigenetic marks and were shown to spread the heterochromatin mark H3K9me2/3 from the insertion sites. 

Considering the influence of dispersed, euchromatic alpha repeats on the expression of neighboring genes, the observed downregulation of all ARs-associated genes upon heat stress as well as differences in the expression of two genes associated with polymorphic alpha satellite repeats suggest a possible contribution of H3K9me3 enriched alpha repeats in gene silencing. It is known that the genome-wide transcriptional response to heat shock in mammals is modulated at promoter-proximal pause, either by increasing or reducing RNA polymerase release, resulting in up or downregulation of many genes [43]. We can exclude the possible influence of this gene regulatory mechanism of heat stress response on *NR3C1* and *ZNF675* genes whose expression is not affected by HS in the absence of associated alpha repeats. However, for other tested genes it is possible that their downregulation listed in Table 1 results not only from H3K9me3 enriched alpha repeats but also from the general transcriptional response to heat shock. Further studies are necessary to resolve contribution of H3K9me3 enrichment at alpha repeats on downregulation of each of these genes. Comparison of expression of a gene with and without particular intronic alpha repeat in the same genetic background (the same cell line) can be helpful in resolving the potential of alpha repeats to modulate gene expression upon heat stress. However, abundant repetitive elements (*Alu*, L1, etc.) adjacent to intronic ARs and to most of intergenic ARs prevented elimination of alpha repeats using CRISPR/Cas9 and creation of modified cell lines suitable for further studies. For intergenic alpha repeats we assumed their association with the closest 5′ and 3′ positioned genes, however because of the 3-dimensional structure of the genome it might be possible that some intergenic repeats are associated with more distant genes and this can be assigned only based on high resolution chromatin interaction maps. The number of genes associated with dispersed alpha repeats is low (46) and gene ontology analysis revealed no significantly enriched pathways or molecular functions within this gene set. It is however interesting that some of the alpha repeat-associated genes are stress or disease-related: *NR3C1* codes for the glucocorticoid receptor which upon binding of stress hormones participates in gene expression regulation [44], *HTRA3* shows drastically downregulated expression in some cancers and it is considered to be a tumor suppressor gene [45,46] while *VAV1* and *PHF20L1* have oncogenic potential [47,48]. In addition, *ZNF675* gene codes for a KRAB zinc-finger protein which specifically targets a subfamily of LTR retrotransposons and is responsible for their silencing [49]. 

The dispersion of satellite DNA repeats throughout the genome is most probably a consequence of the molecular mechanisms of satellite DNA evolution which still need deeper investigation to be fully elucidated [2,50,51]. We propose that some satellite repeat insertions could possibly cause a disease by affecting proper gene expression or by inducing gene mutation, as demonstrated for the insertion of human beta satellite repeats within the splice-acceptor site of a transmembrane serine protease gene [52]. Dispersed satellite repeats could also contribute to genomic instability by mutagenic recombination between these highly homologous elements as shown for recombination between *Alu* repetitive elements which is responsible for human genetic diseases including cancer [53,54]. In addition, euchromatic satellite repeats regulate gene expression, acting as modulators of local chromatin structure upon heat stress [18] or as a source of small RNAs which participate in degradation of maternally inherited transcripts during early embryonic development [55]. A relevant role of satellite DNA in the evolution of species has already been proposed [56,57,58], but further studies are necessary in order to clarify the role of dispersed, euchromatic satellite repeats in gene expression regulation and adaptive evolution, as well as their potential impact on disease.

## Figures and Tables

**Table 1:** The increase of H3K9me3 level at dispersed alpha repeats immediately after heat stress as well as the level of downregulation of alpha repeat-associated genes in different human cell lines. Alpha repeat No, number of monomers within repeat, and similarity to alpha satellite consensus sequence is indicated as well as positions and distance of alpha repeats relative to genes and their distance from TSS. The *p*-values are calculated using the unpaired *t*-test. (- not determined; / not present).

Alpha Repeat No /Number of Monomers / Similarity to Consensus %	The Increase of H3K9me3 (×) after 2 h HS and *p* Values	Alpha Repeat-Associated Gene	Cell Line	Position of Alpha Repeat Relative to Gene and Distance (bp)	Distance of Alpha Repeat from TSS (bp)	The Gene Expression downregulation (×) after1 h/2 h HS and *p* Values
29/0	1.0 (0.211)	*ZNF675*	Cal 27	/	/	-/1.0 (0.059)
29/0	1.0 (0.162)	*ZNF675*	OV-90	/	/	-/1.0 (0.087)
29/0.6 / 88%	1.9 (0.012)	*ZNF675*	MJ90 hTERT	intron	25,978	-/1.7 (0.002)
29/0.6 / 88%	2.1 (0.008)	*ZNF675*	HepG2	intron	25,978	-/1.7 (0.003)
10/1.1 / 86%	2.0 (0.011)	*NR3C1*	MJ90 hTERT	intron	125,971	-/3.5 (0.002)
10/1.1 / 86%	3.2 (0.014)	*NR3C1+allele*	HepG2	intron	125,971	-/2.7 (0.004)
10/0	1.0 (0.158)	*NR3C1-allele*	HepG2	/	/	-/1.0 (0.061)
10/1.1 / 86%	2.1 (0.012)	*NR3C1*	Cal 27	intron	125,971	-/3.7 (0.007)
10/1.1 / 86%	1.7 (0.017)	*NR3C1*	OV-90	intron	125,971	-/2.7 (0.012)
28/1.2 / 70%	3.7 (0.006)	*VAV1*	MJ90 hTERT	intron	25,978	/
28/1.2 / 70%	-	*VAV1*	697	intron	46,492	2.6/- (0.001)
1/0.7 / 82%	4.1 (0.009)	*SLC30A6*	MJ90 hTERT	intron	48,094	-/2.0 (0.003)
14/1.7 / 70%	2.5 (0.011)	*PRIM2*	MJ90 hTERT	intron	59,161	-/2.8 (0.015)
18/0.5 / 76%	4.3 (0.012)	*STAM*	MJ90 hTERT	intron	9163	-/2.0 (0.004)
19/2.4 / 74%	3.6 (0.006)	*PLA2G12B*	MJ90 hTERT	intron	15,104	/
25/1.2 / 88%	3.9 (0.018)	*MYO1E*	MJ90 hTERT	intron	152,899	-/2.3 (0.005)
21/0.7 / 85%	4.0 (0.005)	*DLG2*	MJ90 hTERT	intron	197,351	/
31/1.3 / 71%	2.8 (0.011)	*MAP7*	MJ90 hTERT	intron	15,483	/
3/1.4 / 77%	2.1 (0.012)	*SLC40A1*	MJ90 hTERT	5′ / 38,888	38,888	-/2.0 (0.003)
3/1.4 / 77%	-	*ASNSD1*	MJ90 hTERT	5′ / 46,190	46,190	-/2.3 (0.014)
4/1.4 / 70%	1.9 (0.014)	*ST6GAL1*	MJ90 hTERT	3′ / 29,520	174,981	-/2.1 (0.001)
5/3.4 / 75%	1.8 (0.012)	*HTRA3*	MJ90 hTERT	3′ / 34,942	72,296	-/5.6 (0.011)
5/3.4 / 75%	*-*	*ACOX3*	MJ90 hTERT	3′ / 24,328	98,092	-/1.7 (0.009)
15/2.0 / 86%	2.0 (0.011)	*INTS1*	MJ90 hTERT	5′ / 14,580	14,850	-/2.0 (0.013)
16/0.5 / 72%	2.3 (0.018)	*PHF20L1*	MJ90 hTERT	3′ / 15,772	89,221	-/2.9 (0.010)
17/0.5 / 94%	4.1 (0.011)	*DIP2C*	MJ90 hTERT	5′ / 18,158	18,158	-/1.8 (0.010)
20/1.0 / 86%	2.4 (0.014)	*OR6A2*	MJ90 hTERT	5′/ 14,531	14,531	/
30/1.0 / 70%	1.7 (0.012)	*PPP2R3B*	MJ90 hTERT	5′ / 16,060	16,060	/

## Supplementary Materials

The following are available online at https://www.mdpi.com/2073-4425/11/6/663/s1. Figure S1A: Polymorphism of AR 29 located within intron of *ZNF675* gene among human cell lines; Figure S1B. Sequence of a short amplicon of AR No 29 within intron of *ZNF675* gene; Figure S1C: Sequence of a part of intron of *NR3C1* gene in HepG2 cells encompassing heterozygous AR No 10; Figure S2: Consensus sequence of 171 bp alpha satellite monomer and position of primers used in qPCR; Figure S3: Organization of repetitive sequences identified by RepeatMasker at intronic regions of genes *NR3C1*, *ZNF675,* and *PLA2G12*B; Figure S4: H3K9me3 level at dispersed ARs in HepG2 cells; Figure S5: H3K4me2/3 level at tandemly arranged and dispersed alpha satellite repeats; Figure S6: Dynamics of expression of *VAV1* gene associated with dispersed AR 28 in cell line 697; Table S1: List of primers used for analysis of alpha repeat insertion polymorphism and in ChIP-qPCR experiments; Table S2: List of primers used for expression of alpha repeat-associated genes and of alpha satellite DNA; Table S3: List of alpha satellite repeats dispersed on human chromosomes; Table S4: Expression of genes associated with dispersed alpha satellite repeats after HS in the presence and in the absence of chaetocin.
