# Supplementary material for: Heat Stress Affects H3K9me3 Level at Human Alpha Satellite DNA Repeats
Source: Genes (Basel). 2020 Jun 18;11(6):663. doi: 10.3390/genes11060663 (PMC7348797; doi:10.3390/genes11060663)
Supplement: Supplementary file 1 [file genes-11-00663-s001.pdf]

Heat stress affects H3K9me3 level at human alpha satellite DNA repeats

Isidoro Feliciello[1,2 *], Antonio Sermek[1], Željka Pezer[1], Maja Matulić[3] and Đurđica Ugarković[1*]

[1]Department of Molecular Biology, Ruđer Bošković Institute, Bijenička 54, HR-10000 Zagreb, Croatia

[2] Dipartimento di Medicina Clinica e Chirurgia, Universita' degli Studi di Napoli Federico II, via Pansini 5, I-80131 Napoli, Italy

[3]Department of Biology, Faculty of Science, University of Zagreb, HR-10000 Zagreb, Croatia

***Corresponding authors**: I. Feliciello, phone: +39 3495250441, e-mail: ifelicie@unina.it;

Đ. Ugarković, phone: +385 1 4561197, fax: +385 1 4561177, e-mail: ugarkov@irb.hr;

Figure S1A. Polymorphism of alpha satellite repeat No 29 located within intron of *ZNF675* gene among human cell lines: 1. MJ90 hTERT, 2. 697, 3. MCF-7, 4. HT29, 5. HeLa, 6. Hep2, 7. SW620, 8. SW48 , 9. DLD1, 10. HCT116, 11. ZR75, 12. HepG2, 13. A431, 14. 293T, as well as Cal 27 and OV-90. The AR 29 corresponds to 0.6 of alpha satellite monomer length and is homozygous in all tested cell lines except Cal 27 and OV-90 where it is absent, as revealed by sequencing (Figure S1B). The element No 29 with the flanking region was amplified by PCR using specific primers and the products were separated on agarose gel. Three different DNA isolations from Cal 27 and two from OV-90 cells were tested for the polymorphism of AR 29. M=marker, NC= negative control.

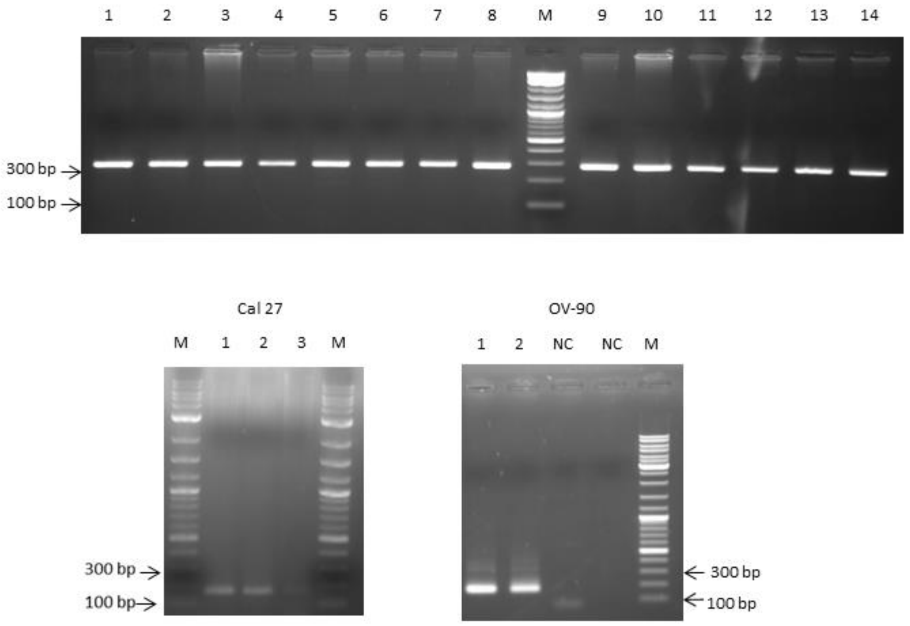

Figure S1B. DNA sequence of a short amplicon within intron of *ZNF675* gene in Cal27 and OV-90 cell line is aligned with the same locus encompassing alpha repeat 29 in MJ90 hTERT cell line. Alpha satellite repeat is marked with red and is completely deleted in Cal27 and OV-90 cells. Position of primers is underlined. *Alu* repeat sequence in which alpha repeat is embedded is marked with blue.

```
ZNF675 MJ90      GCTTACCTTGGCTTCTCAAAGTGCTGGGATTACAGGTGTGAGCCACTATTCCCCGCCCTT
ZNF675 Cal27     GCTTACCTTGGCTTCTCAAAGTGCTGGGATTACAGGTGTGAGCCACTATTCCCAG-----
                 ***************************************************** *

ZNF675 MJ90      TTTTTTTTTCATTTTTCTACACATTTTACTTGTTTGTATTTCTATAGTCTTCTAATGTAG
ZNF675 Cal27     ------TTGCTTTTTTTAAAAAAGTTTTTTT-----------------------------
                       ** * *****  * * * ***  **

ZNF675 MJ90      TCTTGAAATTATAAAGGGCTGGGCATCGGCTCAAGGGCCAGGCACGGTGGCTCACGCCTG
ZNF675Cal27      ----------------------------------GGGTCGGGCCGGGTGGCTCACGCCTG
                                                   *** * ***  ***************

ZNF675 MJ90      TAATCCCAGCACTTTGGGAGGCTGAGGTGGGCGGATCAGGAGGTCAGATAAAAACTAGAA
ZNF675Cal27      TAATCCCAGCACTTTGGGAGGCCGAGGTGGGTAGATCACGAGGTCAG-------------
                 ********************** ********  ***** ********

ZNF675 MJ90      AGAAGCTTTCTGAGAAACTGCTTTGTGATGTGTGCATTCATCTCACAGAGATAAATCTTT
ZNF675Cal27      -----------------------------------------------GAG----------
                                                                ***

ZNF675 MJ90      CTTTTGATTCAGCAGTTTGGAAACCTGGCTAACATGGTGAAACCCGGTGTCTACTA
ZNF675Cal27      --TTCGATAC----------CAGCCTGGCCAACATGGTGAAACCCGGTGTCTACTA
                   ** *** *           * ****** **************************
```

Figure S1C. Sequence of a part of intron of *NR3C1* gene in HepG2 cells encompassing heterozygous alpha satellite element No 10 which is indicated in red. Alpha element is present on a single allele (long) and absent from another one (short). The position of PCR primers is underlined and allele specific primers are additionally marked in yellow and green. *Alu* repeat sequence adjacent to alpha repeat is marked in blue.

```
Short       ACAGGGATATGAAACTCTAACTTATAACTAGTTTCAGGTTTCTTTTTTTTTTTTTTTTT-CCAAGAC
Long        ACAGGGATATGAAACTCTAACTTATAACTAGTTTCAGGTTTCTTTTTTTTTTTTTTTTTTCCAAGAC
            *********************************************************** *******

Short          GGAGTCTTACTCTGTCGCCAAGCTGGAGTACAGTGTTGTGATCTCGGCTCACTGCAACC
Long           GGAGTCTTGCTCTGTCGCCAAGCTGGAGTACAGTGGTGTGATCTCGGCTCACTGCAACC
               *********************************** ***********************

Short          TCTGCCTCCCGGGTTCAAGTGATTCTCCTGCCTCAGCCTCCTGAGTAGCTGGGACTACAG
Long           TCTGCCTCCCGGGTTCAAGTGATTCTCCTGCCTCAGCCTCCTGAGTAGCTGGGACTACAG
               ************************************************************

Short           GTGACACCACCACGCTCCGCTAATTTTTGTATTGTTAGTAGAGACAGGGTTTCACCATG
Long            GTGACACCACCACGCTCCGCTAATTTTTGTGTTTTTAGCAGAGACAGGGTTTCACCATG
                ****************************** ** **** ********************

Short           TTGGCCAGGCTGGTCTCAA-CTCCTGACCTTGTGATCCGCCCACCTCAGCCTCCCAAAAT
Long            TTAGCCAGGATGATCTTGATCTCTTGACCTCGTGATCCGCCTGCCTTGGTCTCCCAAAGT
                ** ****** ** ***  * *** ****** **********  ***  * ******** *

Short           GCTGGGA-----------------------------------------------------
Long            GCGGGGAGACCAAGAAACTTTCTCAGAAACTTCTCACTGTTGTGTGCATTCAGGTCATGG
                ** ****

                Alpha-sat insertion
Long            AGTTGAACGTTTCTTTTGATTGAGCAGTTTTTCAAACACTCATTTTATAGAATCTGCAAG
Long            TGGATATTTGGAGCGCTTTGAGGCCTACGGTGGAAAAGGTAATATCTTCACATAAAAACT
Long            AGACAGAAGGATTCTCACAAACTTCGGGGA

Short           TTATAGGTGTGAGCCATCGTGCCTGGCCCCCTAATATATATCTTATAGGAGTTCCAGAAA
Long            TTACAGGCGTGAGCCACTGCACCCGGCCC---AGGTTTCTTCTT-TAC---TT---GAAA
                *** *** ********  *  ** *****   *  **  * *** **    **   ****


Short           CACCAAAACAAAACCCATCCAGTTCTTCCTT
Long            CACCAAAACAAAACCCATCCAGTTCTTCCTT
               ***********************************
```

Figure S2. Consensus sequence of 171 bp alpha satellite monomer [29] and position of primers used in qPCR.

CATTCTCAGAAACTTCTTTGTGATGTGTGCATTCAACTCACAGAGTTGAACCTTCCTTTTCATAGAGCAGTTTTG

AAACACTCTTTTTGTAGAATCTGCAAGTGGATATTTGGACCGCTTTGAGGCCTACGGTGGAAACGGAAATATCT

TCATATAAAAACTAGACAGAAG

Figure S3: Organization of repetitive sequences at intronic regions of genes *NR3C1*, *ZNF675* and *PLA2G12B*, flanking alpha repeats (ARs) 10, 29 and 19, repectively. ARs are indicated with pink star and regions used for ChIP-qPCR analysis are indicated with red lines. Regions 6 kb distant to ARs are not shown. Repetitive sequences were identified by RepeatMasker and the analysis was performed by UCSC Genome Browser (https://genome.ucsc.edu/).

AR 10

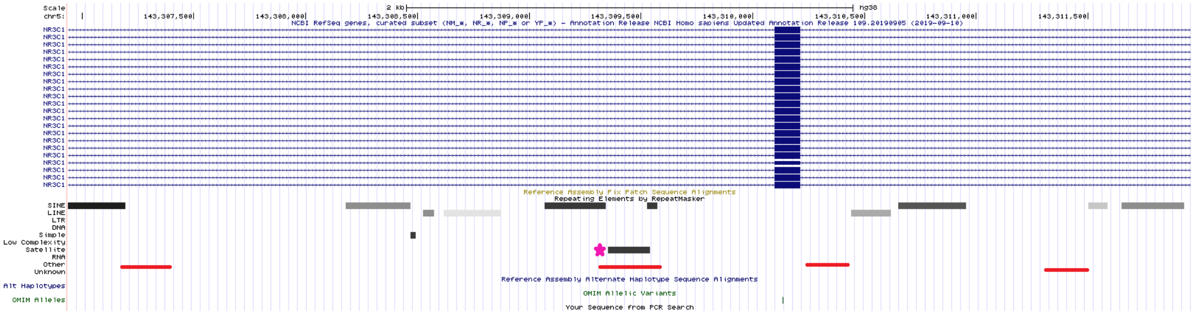

AR 29

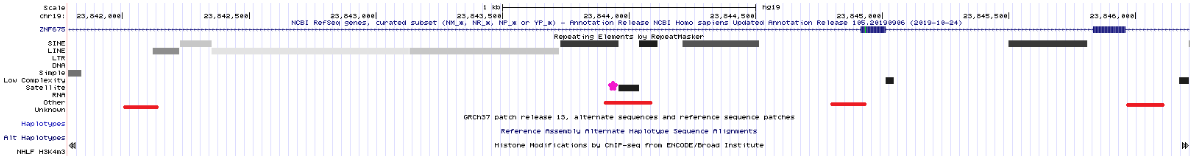

AR 19

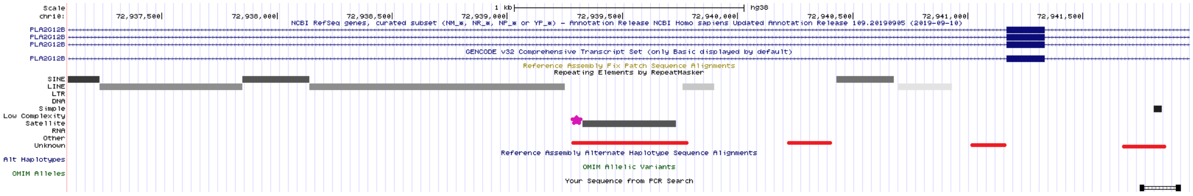

Figure S4. H3K9me3 level in HeG2 cells at intronic alpha satellite repeats (ARs) No 1, 14, 18, 19, 21, 25, 28, 31, and at intronic regions positioned 2-6 kb from each of the AR, either at 5' or 3' site. H3K9me3 levels were measured by ChIP coupled by quantitative real-time PCR at standard conditions (no HS), immediately after 2 hrs of HS (0 min) and at 30 min of recovery (30 min). No value was normalized using No value of input fraction and represents the H3K9me3 level. Columns show average of three independent experiments and error bars indicate the standard deviations.

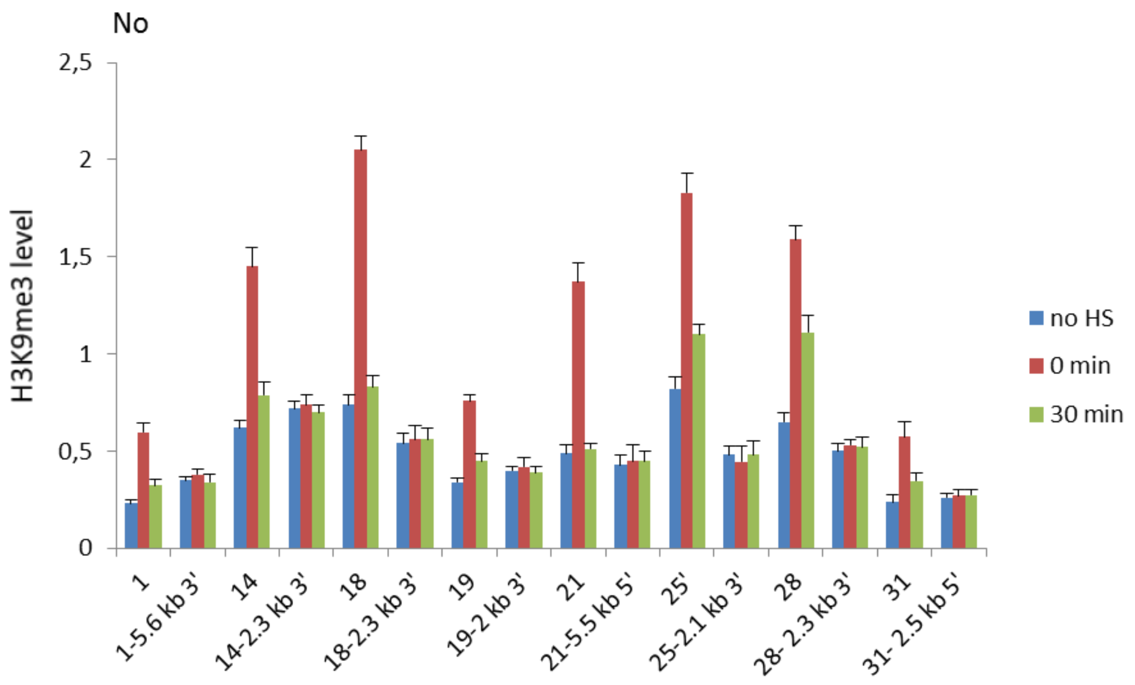

Figure S5. H3K4me2/3 level at tandemly arranged alpha satellite repeats characteristic for heterochromatin (Alpha) and at intronic and intergenic ARs in MJ90 hTERT cells. H3K4me2/3 levels were measured by ChIP coupled by quantitative real-time PCR at standard conditions (no HS), immediately after 2 hrs of HS (0 min) and at 30 min of recovery (30 min). No value was normalized using No value of input fraction. Columns show average of three independent experiments and error bars indicate the standard deviations.

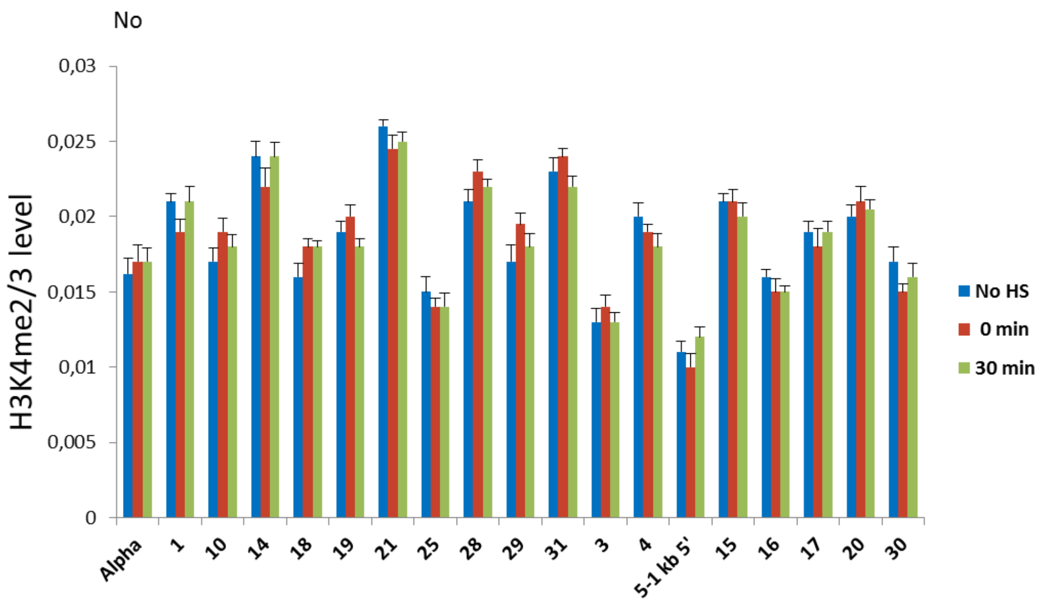

Figure S6. Dynamics of expression of *VAV1* gene associated with dispersed alpha satellite repeat 28 in cell line 697 under standard conditions (no HS), after 1 hr of heat stress (0 min), and at 30 min and 1 hr of recovery. No represents normalized average No value, columns show average of two different qRT-PCR experiments performed in triplicate and error bars represent standard deviations.

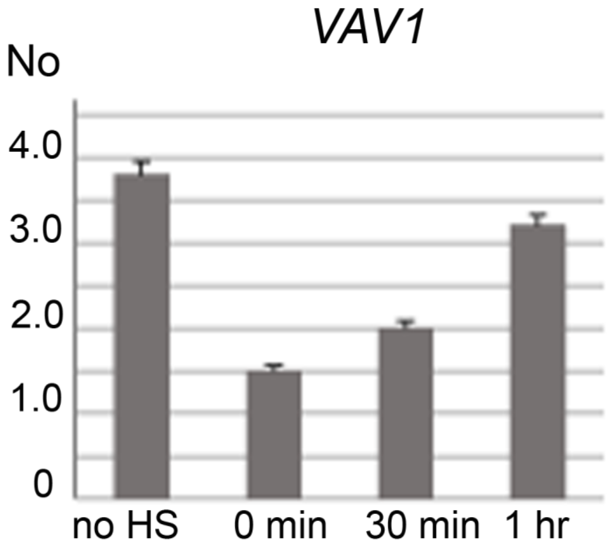

Table S1. List of primers used for analysis of alpha repeat insertion polymorphism and in ChIP-qPCR experiments.

Alpha repeat No – distance from AR	Primers fw	Primers rev
1	GCCTCCTGAGTTCAAGCAAC	GCATGGTGCCTCATTCCTAT
1- 5654 bp 3’	GGATTACAGATGTGAGCCACTG	TGGCATTTAATCTACGGTTGG
2	CCACAAATGGGCCCCTGTTA	GGGAACATCACACTCTGGGG
3	AGGGCTCAGATTTAGGGATTT	AACCCCATAAGGAAGCCAGT
3-2763 bp 3'	GACATTTTATGCCAGAACGGTA	TCTACCTTGGGTTGGCTGTC
4	CCAGACCACGGTAAATGCCA	GACGGATGGCAGAACAAACG
4- 1926 bp 5’	AGCCACATGTTTGAATGAACC	TCCTCAGGAATCCACTATTCG
5	ACAGCAGTGACTCTGAGCCA	AATAGCCTGTTCTTCTGAAGC
5-1045 bp 5’	AGGGGCCATGTTTTCCTCAG	CATCTGGTGGAGGAAGCAGG
5-2427 bp 3’	GGAAGAGCGATTCCAACTCA	TAATGCATGGTGGTGCTGTT
10	ACAGGGATATGAAACTCTAC	AAGGAAGAACTGGATGGG
10- 636 bp 3’	CAATCATTCCTTCCAGCACA	GGCAATACCTGTGGGTGTCT
10- 1778 bp 3’	AATCACCCACTGCTAAAAATGAA	GAAGGTACATTGCCTCTGATCC
10- 1891 bp 5’	GCCTGGGGTCGTCTTCTAGT	ATGAGGGCCTTCTTTCATCA
10- 6276 bp 5’	GGAGCTCCACAGAAGTGTTCA	TTGTGCTTATTTGGGTCTTCACT
13	TAGGAAGTGGGAGAGGGCAA	GACCTGCCCGAAGCTGTTAA
14	GTTCCTGTAAGGGCTCAACG	AGGCTGCAGTAAGCCATGAT
14- 2343 bp 3’	TGCCTCATGGTCTCTCTGTG	TTGTGGGGTTGCAGTTATCA
15	AGTAGTGACTCGTAGGACAC	CTTAGCCCCGTCAAGTTGAC
15-2204 bp 5’	TGAGCAGAGGGATCTTACCC	CTTGGAAGCAAGAGCACCAT
16	GGAGTCAGGCAGGTGAGTTC	CATCACCCCATTGGCTGTCT
16-2232 bp 3’	AGCCTGTTCCCTCCAAAGAT	CCCATCCTGTTTTCTCTGCT
17	ATGGGGAGGTGATGGGGTTA	CCCTCACCAGAATTGCCCTT
17-2310 5’	GAACCTGGTTGAGAATTAGTTGG	TACTTGCCCGTAGGAAATGG
18	TCCCAGTCCATCGAAACCTA	GAAGCTTCATCACCCTCCAA
18- 2291 bp 3’	GGCATGGAGGAGAGTATGGA	TGGAACACTGCAATTCAAAGA
19	CTTGGGCATTTCTGGTCTGT	ATTAGGGCGTTTCACACAGG
19-497 bp 3’	GGTGTGGAGCCAGCTCTTTA	CACCGACTCTATAATTTCTTTTTCC
19-1264 bp 3’	GTGAAGGAGTGCCCAGAATC	GTGCACAGGGAGGTTTGTTC
19- 2046 bp 3’	AAAAATTCAGCCTCCGTTCA	TGGGTGTGTGTGAGATGTTGT
20	TGCTGCCTACAAAAGACCCA	TCTGGGCTCACTGCAATCTC
20-5061 bp 3’	TTTTGCTGCCTAACTGAAACA	TGAAGGACACAATTCACACCA
21	GCACCACACTGGTCTTCC	CTGTATTTCAGCATGAGTGACAG
21-5450 kb 5’	CCAATGTTTTTGAGGTTATGG	TTGGTGTACGTTTAAGAAAGTAGGA
25	CGACATGGGTCCAGTCTGAT	AGGAATCTGGATATGTCTTCCA
25- 2096 bp 3’	ACTCCCTTCCCTGGTAAACA	CATCCCACTCATTGCTGACA
28	AGACTCCATCCCCCTCAAAA	TCAAGGTGTCAGTAGGGTTGG
28- 2260 bp 3’	ACACCCTGTCTGCTCTGTCC	TGAGCACTCACTCGATCTGG
29	GCTTACCTTGGCTTCTCAAAGT	TAGTAGACACCGGGTTTCACC

29- 625 bp 3’	AAGCGGACACTGGTTCTCAC	GGGAAGCCGTGTTTTAAAGG
29- 1844 bp 5’	CAAAATGGGTGCAAATCATAGA	CCATTTTCTGTTTCAATTTTGTCT
29- 1890 bp 3’	GGCCACATCCCTAAATGTCA	CTGGTGTTGTGGCTCTTGTG
29- 6153 bp 5’	CAGGTGAGCACAATGCAAAG	TGCTGGGAGTACAGGTGTGA
30	CCTGAGTGGGGAGAAAGCTG	CTGTAGGCCCAGCTACAAGG
30 -2250 bp 5’	GTTGCGGGACCTTCCTCT	CAGCTGCCCTCCAAAAATAG
31	CGCTAATGCTGAAGACATGC	GAAGTGGAATGGGATCTGAAA
31- 2425 bp 5’	AATGTTGAGCGGAGATCCAG	CTCTGCCTGCTGTGTTTGAG

Table S2. List of primers used for expression of alpha repeat-associated genes and of alpha satellite DNA.

**Gene**	**Primers fw**	**Primers Rev**
SLC30A6	TGATCTTGCTGGAGCATTTG	AAACATGGGGTGGTGTTGTCT
TGFA	TGTTCGCTCTGGGTATTGTG	ACGTACCCAGAATGGCAGAC
SLC40A1	AACAAGCACCTCAGCGAGAG	CACATCCGATCTCCCCAAGT
ASNSD1	CACTGGAATTGGTGCAGATG	GTTTGCTTTTTCCCAAATCG
ST6GAL1	TGGGATGCTTGGTATCATCA	GTGGCTTTTCCAAGCAGGTA
RPL392	GAGGCGTCTCGGAGTCTCA	AAATAAAATCGGCGCCCTGG
HTRA3	GAGGCCACCATCAAAGACAT	GGCATCCGTCTGGATGTAG
ACOX3	TCCCAACTGCACATACGAAG	CAGGTAGCAAACCAGCCACT
NR3C1	AGGCTGGAATGAACCTGGAA	AACAGTGACACCAGGGTAGG
NR3C1- HepG2-	CACCTCAGCCTCCCAAAATGCTGGGATTAT	AAGGAAGAACTGGATGGG
NR3C1- HepG2+	TGCCTTGGTCTCCCAAAGTGCGGGGAGACC	AAGGAAGAACTGGATGGG
FAM83B	TCAGTTCAGCGTCTCAGGAA	ACCATGCTGAGGTGAGCTTT
PRIM2	TGGACTTAAGTTGGGGTTCG	CAAAGCCTTGGACAGTTTGG
INTS1	GTGTCTGTGCTGGCCTGTAA	CTGGATGTTGTCCACAAACG
MAFK	CGACTAATCCCAAACCGAAT	CTCCAGCTCCTCCTTCTGC
PHF20L1	CTGCAGCCAAGAACAAAACA	TCTCTGGCTGTGGAAAGACC
TG	TCTATCAGAGACGCCGCTTT	TTCGAGATTTCTCGCAGGTT
DIP2C	CCAACCCAGAAACAAAAGGA	AGGAACCCCAAGTAGCCTGT
STAM	AACAAAGGCAGCAGTCAACC	TTGATGGGTTTCACCTTTCC
PLA2G12B	GCAAAATTCCGATGGTGTCT	TTCCTTCTCCTCCTCTGCAC
OR6A2	TGATGCACATTCCTTCGGCT	GCGCAGGCAGTAAATGATGG
OR10A5	CTACGTGCTCCTCACACCTC	GGGCTAGGACCTTGTGGAAG
DLG2	TTGCATGTTACTGTGCACTCC	CAGAGGAGAAATATGAGACTGCAA
MYO1E	CTGGGAGGAAAGCAGGGTAA	ACACTTTACTCCTCCCCAGC
ZNF675	ACACTGCACAGCGGAATTTA	GGGGTTCATTCACCATCTCA
VAV1	TGCTTCAAGTCTCTGGACACCAC	TCTCGGGCGCAGAAGTCATA
PPP2R3B	AGCTGACCGAATTCTTCTCG	TTTTCTGCCTCGTGTGACTG
MAP7D2	TTCCTGTTGTGAACTTCGGG	CCTTCCCTTTCCTTGTTGCT
Alpha satellite	TGTGTGCATTCAACTCACAGAG	TGCACACATCACAAAGAAGTTT
GUSB	GAAAATATGTGGTTGGAGAGCTCATT	CCGAGTGAAGATCCCCTTTTTA

Table S3. List of alpha satellite repeats dispersed on human chromosomes. Genomic positions of repeats (genome assembly GRCh38/hg38), their composition and similarity to consensus alpha satellite sequence is indicated as well as genes associated with each repeat. Alpha satellite elements tested for polymorphism and their associated genes used in expresion analyses are indicated in bold.

**chr No**	**alpha repeat No**	**Genome position**	**Similarity to alpha satellite consensus/size**	**associated gene**
2	**1**	**32213935-32214051**	**82%, 0.7 monomer**	**solute carrier family 30 member 6, (SLC30A6 ID: 55676), intron**
	**2**	**70431412-70431596**	**88%, 0.9 monomer**	130438 bp at 5' side: protein FAM136A isoform X1, ID: 84908
				**19263 bp at 3' side: TGFA transforming growth factor alpha isoform 2 preproprotein, (TGFA ID: 7039)**
	**3**	**189619699-189619943**	**77%, 1.4 monomers**	**38888 bp at 5' side: solute carrier family 40 member 1 isoform X1, (SLC40A1 ID: 30061)**
				**46190 bp at 3' side: asparagine synthetase domain-containing protein 1 isoform X1, (ASNSD1 ID: 54529)**
3	**4**	**187105323-187105556**	**70%, 1.4 monomers**	**29520 bp at 5' side: beta-galactoside alpha-2,6-sialyltransferase 1 isoform b, (ST6GAL1 ID:6480)**
				**15589 bp at 3' side: 60S ribosomal protein L39-like, (RPL392 ID: 116832)**
4	**5**	**8342053-8342633**	**75%, 3.4 monomers**	**34942 bp at 5' side: HtrA serine peptidase 3, (HTRA3 ID: 94031)**
				**24328 bp at 3' side: peroxisomal acyl-coenzyme A oxidase 3 isoform X4, (ACOX3 ID: 8310)**
	6	77489446-77489557	82%, 0.7 monomer	323522 bp at 5' side: cyclin-G2 isoform X1, ID:901
				116309 bp at 3' side: C-X-C motif chemokine 13 precursor ID: 10563
	7	111490801-111491006	78%, 1.2 monomer	857833 bp at 5' side: PITX2 paired like homeodomain 2:, ID: 5308
				335840 bp at 3' side: ncRNA, ID: 105377368
	8	135576694-135576783	89%, 0.52 monomer	1375501 bp at 5' side: polyadenylate-binding protein 4-like, ID: 132430
				1944246 bp at 3' side: protocadherin-18 isoform 2 precursor, ID: 54510
5	9	100196524-100196622	89%, 0.6 monomer	671163 bp at 5' side: putative POM121-like protein 1-like isoform X2, ID:100652833

				192353 bp at 3' side: putative POM121-like protein 1-like, ID:441098
	**10**	**143309356-143309541**	**88%, 1.1 monomers**	**nuclear receptor subfamily 3 group C member 1(NR3C1, ID: 2908), intron**
	11	161040048-161040173	73%, 0.73 monomer	354828 bp at 5' side: probable phospholipid-transporting ATPase VB isoform X3; ID: 23120
				253908 bp at 3' side: gamma-aminobutyric acid receptor subunit beta-2 isoform 2, ID:2561
	12	161163077-161163193	70%, 0.68 monomer	477857 bp at 5' side: probable phospholipid-transporting ATPase VB isoform X3, ID: 23120
				130888 bp at 3' side: gamma-aminobutyric acid receptor subunit beta-2 isoform 2, ID:2561
6	**13**	**54991256-54991369**	**74%, 0.7 monomer**	**49249 bp at 5' side: protein FAM83B isoform X1; (FAM83B ID: 222584)**
				183219 bp at 3' side: orexin receptor type 2; ID: 3062
	**14**	**57377750-57378048**	**70%, 1.7 monomer**	**DNA primase subunit 2 (PRIM2 ID: 5558) intron**
7	**15**	**1519232-1519571**	**86%, 2 monomers**	**14850 bp at 5' side: integrator complex subunit 1 (INTS ID: 26173)**
				**19622 bp at 3' side: transcription factor MafK, (MAFK ID: 7975)**
8	**16**	**132864579-132864640**	**72%, 0.5 monomer**	**15772 bp at 5' side: PHD finger protein 20-like protein 1 isoform X10; (PHF20L1 ID: 51105)**
				**2361 bp at 3' side: thyroglobulin precursor, (TG ID: 7038)**
10	**17**	**707868-707956**	**94%, 0.5 monomer**	**18158 bp at 5' side: DIP2C disco interacting protein 2 homolog C; (DIP2C ID: 22982)**
				104970 bp at 3' side: la-related protein 4B isoform X2; ID: 23185
	**18**	**17653288-17653378**	**76%, 0.5 monomer**	**signal transducing adapter molecule 1; (STAM ID: 8027), intron**
	**19**	**72939326-72939731**	**74%, 2.4 monomer**	**phospholipase A2 group XIIB; (PLA2G12B, ID: 84647), intron**
11	**20**	**6810439-6810612**	**86%, 1 monomer**	**14531 bp at 5' side: olfactory receptor 6A2, (OR6A2 ID:338755)**
				**35071 bp at 3' side: olfactory receptor 10A5, (OR10A5 ID: 338755)**
	**21**	**85431074-85431184**	**85%, 0.7 monomer**	**discs large homolog 2, (DLG2 ID: 1740), intron**
13	22	23423970-23424059	71%, 0.5 monomer	12731 bp at 5' side: sacsin isoform X2; ID: 26278
				166125 bp at 3' side: tumor necrosis factor receptor superfamily member 19 isof; ID: 55504
	23	23424792-23424872	71%, 0.5 monomer	13553 bp at 5' side: sacsin isoform X2, ID: 26278

				165312 bp at 3' side: tumor necrosis factor receptor superfamily member 19 isof., ID: 55504
15	24	24754024-24754329	70%, 1.8 monomers	74686 bp at 5' side: nuclear pore-associated protein 1, ID: 23742
				200720 bp at 3' side: SNRPN upstream reading frame protein,ID: 6638
	**25**	**59219764-59219973**	**88%, 1.2 monomers**	**myosin IE (MYO1E, ID: 4643), intron**
	26	93907560-93907673	79%, 0.7 monomer	700821 bp at 5' side: putative uncharacterized protein UNQ9370/PRO34162; ID: 105370980
				390593 bp at 3' side: MCTP2 multiple C2 domains, transmembrane 2, ID: 55784
	27	93907750-93907849	77%, 0.6 monomer	701011 bp at 5' side: putative uncharacterized protein UNQ9370/PRO34162, ID: 105370980
				390417 bp at 3' side: multiple C2 and transmembrane domain-containing protein 2, ID: 55784
19	**28**	**6819160-6819369**	**70%, 1.2 monomers**	**vav guanine nucleotide exchange factor 1, (VAV, ID: 7409), intron**
	**29**	**23661142-23661242**	**86%, 0.6 monomer**	**zinc finger protein 675, (ZNF675, ID: 171392), intron**
X	**30**	**402751-402878**	**70%, 1 monomer**	**16060 bp at 5' side: PPP2R3B protein phosphatase 2 regulatory subunit B''beta, (PPP2R3B ID: 28227)**
				228020 bp at 3' side: short stature homeobox protein isoform SHOXb, ID: 6473
	**31**	**20101282-20101513**	**71%, 1.3 monomers**	**MAP7 domain containing 2 (MAP7D2, ID: 256714), intron**

Table S4. Expression of genes associated with dispersed alpha satellite repeats after HS in the presence and in the absence of chaetocin, inhibitor of histone methyltransferase Suv39H1. The expression is monitored immediately after heat stress (HS) and the level of upregulation relative to the control without chaetocin is shown. The P-values are calculated using the unpaired t-test.

Gene name	Cell line	The level of upregulation (X) after chaetocin and P values
ZNF675	MJ90 hTERT	1.50 (0.012)
NR3C1	MJ90 hTERT	1.61 (0.013)
SLC30A6	MJ90 hTERT	1.62 (0.008)
HTRA3	MJ90 hTERT	1.32 (0.009)
PRIM	MJ90 hTERT	1.31 (0.011)
INTS1	MJ90 hTERT	1.50 (0.007)
STAM	MJ90 hTERT	1.30 (0.008)
MYO1E	MJ90 hTERT	1.81 (0.011)
SLC40A1	MJ90 hTERT	2.00 (0.013)
ASNSD1	MJ90 hTERT	2.30 (0.005)
ST6GAL1	MJ90 hTERT	2.08 (0.007)
ACOX3	MJ90 hTERT	1.70 (0.013)
PHF20L1	MJ90 hTERT	1.30 (0.009)
DIP2C	MJ90 hTERT	1.55 (0.008)
ZNF675	Cal 27	0.95 (0.176)
NR3C1	Cal 27	1.60 (0.011)
ZNF675	OV-90	1.00 (0.122)
NR3C1	OV-90	1.70 (0.010)
ZNF675	HepG2	1.55 (0.013)
NR3C1 - alpha	HepG2	1.05 (0.085)
NR3C1+ alpha	HepG2	1.70 (0.015)
